# Effectiveness of Artificial Intelligence–Based Platform in Administering Therapies for Children With Autism Spectrum Disorder: 12-Month Observational Study

**DOI:** 10.2196/70589

**Published:** 2025-04-28

**Authors:** Harini Atturu, Somasekhar Naraganti, Bugatha Rajvir Rao

**Affiliations:** 1 Psychiatry, CARE Hospitals, Hyderabad, India; 2 Department of Psychology, Rainbow Children's Hospital, Hyderabad, India; 3 CognitiveBotics Technologies Private Limited, 6th Floor, TSIIC, MYHOME TWITZA, Raidurg, Telangana, Hyderabad, 500081, India, 91 7893855499

**Keywords:** autism spectrum disorder, neurodevelopmental disorders, applied behavior analysis, software, artificial intelligence

## Abstract

**Background:**

A 12-month longitudinal observational study was conducted on 43 children aged 2‐18 years to evaluate the effectiveness of the CognitiveBotics artificial intelligence (AI)–based platform in conjunction with continuous therapy in improving therapeutic outcomes for children with autism spectrum disorder (ASD).

**Objective:**

This study evaluates the CognitiveBotics software’s effectiveness in supporting children with ASD through structured, technology-assisted learning. The primary objectives include assessing user engagement, tracking progress, and measuring efficacy using standardized clinical assessments.

**Methods:**

A 12-month observational study was conducted on children diagnosed with ASD using the CognitiveBotics AI-based platform. Standardized assessments, include the Childhood Autism Rating Scale (CARS), Vineland Social Maturity Scale, Developmental Screening Test, and Receptive Expressive Emergent Language Test (REEL), were conducted at baseline (T1) and at the endpoint (T2). All participants meeting the inclusion criteria were provided access to the platform and received standard therapy. Participants who consistently adhered to platform use as per the study protocol were classified as the intervention group, while those who did not maintain continuous platform use were designated as the control group. Additionally, caregivers received structured training, including web-based parent teaching sessions, reinforcement strategy training, and home-based activity guidance.

**Results:**

Participants in the intervention group demonstrated statistically significant improvements across multiple scales. CARS scores reduced from 33.41 (SD 1.89) at T1 to 28.34 (SD 3.80) at T2 (*P*<.001). Social age increased from 22.80 (SD 7.33) to 35.76 (SD 9.09; mean change: 12.96, 56.84% increase; *P*<.001). Social quotient increased from 53.26 (SD 11.84) to 64.75 (SD 16.12; mean change: 11.49, 21.57% increase; *P*<.001). Developmental age showed an improvement from 30.93 (SD 9.91) to 45.31 (SD 11.20; mean change: 14.38, 46.49% increase; *P*<.001), while developmental quotient increased from 70.94 (SD 10.95) to 81.33 (SD 16.85; mean change: 10.39, 14.65% increase; *P*<.001). REEL scores showed substantial improvements, with receptive language increasing by 56.22% (*P*<.001) and expressive language by 59.93% (*P*<.001). In the control group, while most psychometric parameters showed some improvements, they were not statistically significant. CARS scores decreased by 10.62% (*P*=.06), social age increased by 52.27% (*P*=.06), social quotient increased by 19.62% (*P*=.12), developmental age increased by 44.88% (*P*=.06), and developmental quotient increased by 11.23% (*P*=.19). REEL receptive and expressive language increased by 34.69% (*P*=.10) and 40.48% (*P*=.054), respectively.

**Conclusions:**

Overall, the platform was an effective supplement in enhancing therapeutic outcomes for children with ASD. This platform holds promise as a valuable tool for augmenting ASD therapies across cognitive, social, and developmental domains. Future development should prioritize expanding the product’s accessibility across various languages, ensuring cultural sensitivity and enhancing user-friendliness.

## Introduction

Autism, otherwise known as autism spectrum disorder (ASD), is a neurodevelopmental disorder with a wide continuum of associated cognitive and neurobehavioral deficits including, but not limited to, 3 core defining features: impairments in social interaction and impairments in verbal and nonverbal communication, combined with restricted and repetitive patterns of behaviors [1]. Such impairments can impede an individual’s social level of interaction, learning aptitude, and employability, leading to poor long-term outcomes, difficulties in socializing, poor job performance, and difficulties in activities of daily living [2-5]. The estimated prevalence of ASD has increased from 1 in 10,000 in the 1960s to at least 1 in 36 today [6,7].

**Figure 1.**
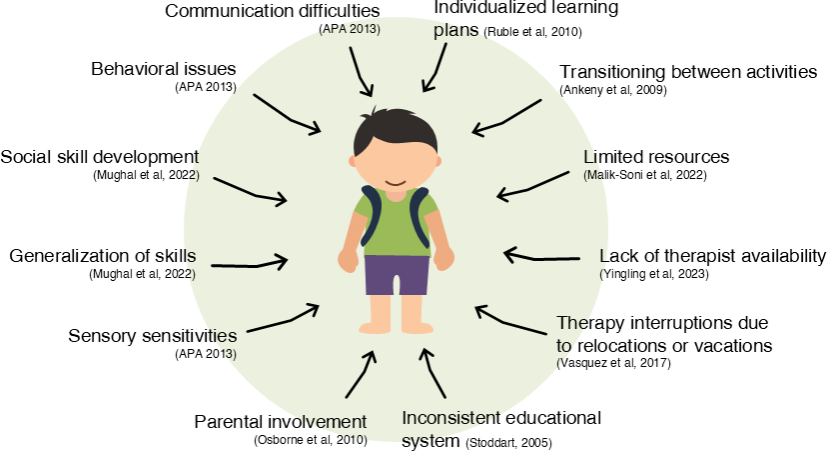
Challenges in providing therapy to individuals with autism spectrum disorder [13-20].

The cause for the rise of children diagnosed with ASD is unknown [8]. What is clear is that early and consistent intervention is crucial for positive long-term outcomes [9]. Currently, there are no medical treatments that can effectively cure individuals with ASD, with most interventions involving applied behavioral analysis (ABA), speech and language therapy, and sensory integration to address the core symptoms of ASD [10,11]. To provide adequate and quality therapy to children with autism, a team of trained professionals ranging from pediatricians, child psychiatrists; occupational, behavioral, and speech therapists; psychologists, specialist teachers, and dedicated caregivers are necessary [12]. Providing therapy to children with autism can be rewarding but challenging due to several factors. Figure 1 provides an insight into the challenges faced by the stakeholders in the care and support of children with autism [13-20].

As is, the solution to many of today’s challenges may be the leveraging of cutting-edge technologies to enhance autism intervention; these technologies include the use of machine learning, deep learning in artificial intelligence (AI), animated gaming, and data analytics. Computer-assisted interventions (CAIs) are particularly appealing to underresourced schools due to the potential to provide cost-effective individualized instruction and allow teachers to offer concurrent group instruction. Several available CAIs have integrated evidence-based interventions and complement current therapies for individuals with ASD [21].

Research suggests that CAIs, when applied effectively, can enhance learning by fostering four key components of the learning process: (1) active engagement, (2) group participation, (3) regular interaction and feedback, and (4) integration with real-life settings [22]. Furthermore, the convenient access of CAIs among parents and therapists allows ease of access to these technologies right in the palm of their hands [23]. During the recent COVID-19 pandemic, there was significant disruption and reduction in conventional therapies. As a means to continue therapy, many therapists sought to use CAIs, leading to a jump in usage from 15% to 61% [24].

Through the use of intelligent systems–based AI technologies, therapists and parents alike can provide supplementary and consistent therapy to individuals with ASD and enhance outcomes [25-28]. In 2 recent articles, the prospect of integrating AI into standard practices for autism therapy has great potential to improve social and communication outcomes in individuals with autism [29,30].

The integration of video modeling in ABA allows the individual to observe a recorded video of a specific task, gradually enabling independent performance by clearly presenting the instructions and essential stimuli needed to complete the task. Several studies have demonstrated the effectiveness of this strategy across various complex social tasks, such as acquiring conversational skills, commenting, complimenting, and enhancing pragmatic abilities, as well as initiating and maintaining social relationships [31].

Gaming systems provide a sensory stimulus, where numerous studies have found an attraction factor for participation through a framework or application that provides additional animation and images [32,33]. AI-driven games can improve cognitive skills, social interaction, and emotional regulation. Such games can be modified to the specific needs of individuals with autism, offering personalized learning objectives. Studies have suggested that integrating AI-based interventions into standard therapy can improve the behavioral patterns of children with autism [34,35]. Animation games use engaging animated characters and scenarios to teach essential skills, making learning enjoyable and less stressful for children with autism, thus improving their attention span and resulting in a greater retention of learned skills. Studies using animation-based interventions have observed significant improvements in language acquisition and social skills [36,37]. All these technology-driven solutions have been shown to significantly enhance outcomes and bridge the limitations of therapists and parents in managing challenging behaviors among children with ASD.

As a result, CognitiveBotics, an AI-powered assistive technology, was designed and developed. The platform allows children with autism and their parents and therapists to effortlessly access its program anytime, anywhere, since it only requires a gadget (eg, a laptop or tablet) and access to an internet connection. The development process involved a multidisciplinary approach, combining insights from clinical psychology, child development, and technology experts. The platform provides a “digital” VARK (visual, auditory, read/write, and kinaesthetic) opportunity range to help children acquire social, communication, emotional, and behavioral skills, while automatically recording progress for therapists [38]. For parents, the platform is an easy-to-use digital tool offering training sessions on strategies and techniques, ensuring continuity of therapy at home. For further information on the platform, visit [39].

During the COVID-19 pandemic, a survey was conducted among therapists working with children diagnosed with ASD. Due to the reduction in conventional therapies, the therapists observed a moderate to severe impact on individuals’ learning (73%), while parents were impacted emotionally and psychologically (85%). Before the pandemic, only 22% of therapists expressed a willingness to use any digital technology in autism intervention, however, this number tripled to 65% due to the constraints imposed by the lockdown [40]. There was an urgent need for standardizing digital health technologies that can be parent-mediated [41]. An initial pilot study was conducted between November 2020 and April 2021 to assess the software’s capabilities using a set of 19 different skills. Throughout the study, the software effectively collected and recorded data during the user interaction, demonstrating its effectiveness in real-time data collecting and analysis [40].

Subsequently, to further evaluate the effectiveness of the CognitiveBotics AI-based platform in augmenting therapies for individuals with ASD, an observational, longitudinal study with an adequate sample size was conducted to assess different domains—the social/emotional, language/communication, and cognitive development of individuals who used the platform for 12 months. The initial study revealed minor glitches, which were promptly addressed, and parents of the individuals expressed a willingness to continue using the app, highlighting its potential impact.

## Methods

### Overview

The observational, longitudinal study was designed to evaluate the effectiveness of the CognitiveBotics AI-based software over a 12-month period. By understanding the practical challenges and assessing the software’s effectiveness, the study provides a foundation for the future development and design of a trial.

The primary objectives of the study are as follows:

1. User engagement: assess the ability of both children and parents to effectively use the software and follow web-based instructions.
2. Progress tracking: evaluate the software’s capability to automatically log the child’s daily progress and provide visual graphical feedback on the dashboard.
3. Efficacy measurement: using established clinical parameters to evaluate progress at T1 and T2 across multiple measures.

### Scoring Systems

Qualified therapists conducted assessments at baseline and at a 1-year follow-up, using the following specific parameters to evaluate progress over time.

The Childhood Autism Rating Scale (CARS) score is a factor analysis–based scale used for assessing the presence and severity of symptoms of autism spectrum disorders [42]. Scores between 30 and 37 are considered as mild to moderate autism and scores between 38 and 60 are considered as a severe level of autism. According to Russell et al [43], CARS has an acceptable level of sensitivity and specificity in Indian populations.

The Vineland Social Maturity Scale (VSMS) scores were compared between groups, assessing changes in social age (SA) and social quotient (SQ). This scale has been used to measure the adaptive behaviors of children with or without ASD by measuring their developmental profile in 8 domains and scoring SA and SQ. Originally developed by Doll in 1935 [44], VSMS was adapted by Malin in 1956 [45] to better suit the Indian population, ensuring its cultural relevance and applicability. This adaptation was further modified by Bharatraj in 1992, incorporating additional changes [46].

The Developmental Screening Test (DST), which measures developmental age (DA) and developmental quotient (DQ), assesses the developmental progress of children across various domains, including motor skills, language, social behavior, and cognitive abilities. It helps in determining the DA and DQ of the participants, which reflects their level of functioning in comparison to typical developmental milestones [47]. Recognizing that many developmental assessments at that time were standardized on Western populations, in 1977, Bharatraj adapted the DST to be more sensitive to the developmental norms of Indian children [48].

The Receptive and Expressive Emergent Language (REEL) test is designed to identify infants and toddlers who have language impairments or who have other disabilities that affect language development. It has 2 core subtests, receptive language age (RLA) and expressive language age (ELA), which are based on caregiver reports and converted into age-equivalent scores. A study conducted with Hindi-speaking children found the REEL assessment to be valid, reliable, and effective in assessing language outcomes [49].

### Recruitment

Recruitment for the study took place from January to April 2023 and the completion of the study was 12 months after the last participant was recruited. Parents whose children were diagnosed with ASD and attending Rainbow Hospital in India were identified by the clinical team. Recognizing that individuals with ASD may have a higher chronological age but a lower social or developmental age, participants were accepted if their social or developmental age was between 2 and 18 years. The parent information sheet regarding the study was provided to all identified parents. Parents who expressed interest in their child’s participation were contacted by the principal investigator’s team. Textbox 1 shows the inclusion, exclusion, and withdrawal criteria of the study.

Textbox 1. **Inclusion, exclusion, and withdrawal criteria for participants.**

**Inclusion criteria**

Children who met all the following inclusion criteria were enrolled in the study:

1. Children diagnosed with autism spectrum disorder using assessment scales such as the Childhood Autism Rating Scale.

2. Children aged between 2 and 18 years.

3. Children with associated comorbidities were included on the condition that the child can use the platform.

4. Children with the ability to understand and respond to instructions given in English.

5. Children with access to a device on which the software can be accessed using an internet connection.

6. Children with parents who consented for their child to use the software.

**Exclusion criteria**

1. Children with parents who were not willing to consent to the study.

2. Children without access to a tablet, computer, or internet connection.

3. Children unable to understand English.

**Withdrawal criteria (removal of participants from the therapy or assessment)**

Any participant was allowed to voluntarily discontinue participation in the study at any time after giving informed consent and before the completion of the last visit of the study. This would not affect the care provided by their clinical team. The reasons for participant withdrawal were recorded and included but were not limited to the following:

1. Participant was no longer willing to continue in the study.

2. Study termination by sponsor or independent ethics committee.

3. Investigator’s discretion (for safety reasons).

When a participant withdrew from the study, the investigator clearly documented the reason in the medical records and completed the appropriate case report form describing the reason for discontinuation. In addition, every effort was made to complete the appropriate assessment.

During this stage, the study objectives and procedures were thoroughly explained, and any questions from the parents were addressed. Informed consent was obtained from those who agreed to participate, and documentation was appropriately maintained. At baseline, clinical assessments including the CARS, DST, VSMS, and REELs were administered. Parent training sessions, conducted either online or offline, were arranged to familiarize parents with the platform and its usage. Parents who had training were granted access to the software and instructed to ensure their children used the software for at least 20 minutes per session, with a minimum of 3 sessions per day over 12 months, followed by home-based activities to reinforce learning. At the beginning of the study, we requested parents to use the software in addition to the standard care they were providing to their children and for ethical reasons did not ask them to stop any other treatments or therapies.

Participants were scheduled for 3 visits during the active study period:

- Visit 1 (day 0, T1): baseline clinical assessments were conducted.
- Visit 2 (6 months): clinical parameters were reassessed.
- Visit 3 (12 months, T2): final clinical assessments were conducted.
- Data from the software tracking the child’s progress were collected for statistical analysis at each stage.

Additionally, a follow-up phone call was made every 15 days between the physical visits to verify the child’s regular usage of the software and address any concerns. This telephonic follow-up ensured adherence to the study protocol and provided support for parents throughout the trial.

### Software-Delivered Program

Using tablets or a computer, the platform offers evidence-based therapeutic interventions through a high-quality, patented software program that addresses a broad spectrum of learning difficulties by teaching small, key behaviors incrementally. This aims to improve learning outcomes and developmental progress in individuals with ASD by providing a comprehensive digital platform that supports various learning styles and therapeutic needs. It is designed to personalize learning, adjust difficulty levels, and provide real-time feedback and support to both parents and children.

Upon initially using the platform, parents were registered in the system and requested to complete an auto-generated individualized learning plan (ILP) questionnaire generated by the software. This enabled the software to ascertain the child’s current developmental state and learning needs. If there were any difficulties or queries from the parents regarding the questionnaire, a study coordinator was available to assist with the onboarding process. Parents were then requested to attend a webinar session, where an interactive orientation on the software and its features was given, and any queries were addressed. Additionally, parents received a user manual and a navigation video for reference. Participation in this webinar session was mandatory before an ILP was assigned to the child.

Based on the parental responses and child assessments, an ILP consisting of 3 target goals was generated by AI models focusing on 4 domains (social/emotional, language/communication, cognitive, and movement/physical development). Table 1 contains the lesson plan within the software and its advantages in providing adjunct therapy to children with ASD. The content is personalized and mapped to individual learning objectives, guided by therapist-defined developmental goals.

**Table 1.** Lesson plan structure and associated advantages of the platform.

Goal/skill domain	Task/learning objective	Methodology and advantages
Eye contact/attention	Looking at the object	Gamified, visually engaging content designed for children with neurodiverse profiles. Encourages sustained visual attention through interactive elements.
Eye contact/attention	Responding to name	Multimodal cues and visual prompts enhance auditory responsiveness and social awareness.
Imitation skills	Imitating arm, leg, or facial movements	Structured video models guide imitation in a low-anxiety, judgment-free digital space.
Cognitive skills	Number identification, shape recognition	Tasks scaffold foundational academic concepts in a playful, exploratory manner.
Communication/language	Labeling objects, requesting help	Activities promote expressive and receptive communication. Coviewing with caregivers enhances language modeling.

**Figure 2.**
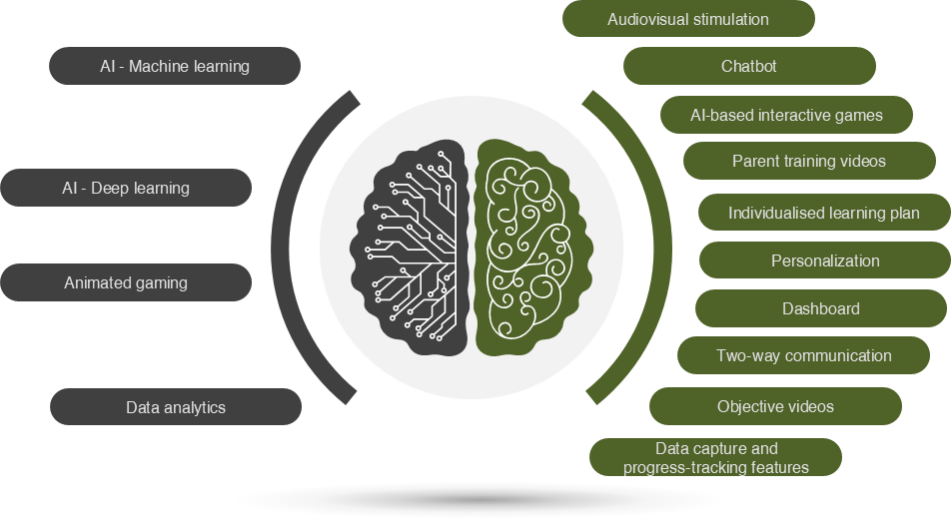
The technologies and features in the software. AI: artificial intelligence.

Before engaging in any lessons, parents were encouraged to watch the objective videos to improve the reasoning of mastering each goal. A practice session was available for skill reinforcement; however, the scores in these practice sessions were not recorded for progression to the next stage. Each daily practice session lasted 20 minutes, after which the software automatically concluded the learning session and redirected the child to the dashboard. If the caregiver determined that the child was prepared for an additional session, they had the option to initiate a new session., Overall, there are 227 activities or tasks organized under goals. Figure 2 presents the technologies and features of the CognitiveBotics platform.

The session begins with the caregiver launching the daily schedule on the CognitiveBotics app. This schedule presents a sequence of personalized tasks aligned with the child’s developmental goals. Each task is supported by engaging, gamified digital content designed specifically for children with ASD. Caregivers are encouraged to coview and participate in the learning process, fostering emotional bonding and reinforcing engagement through shared experience. Alternatively, under parent supervision, the child may explore the content independently, depending on their comfort and developmental level.

**Figure 3.**
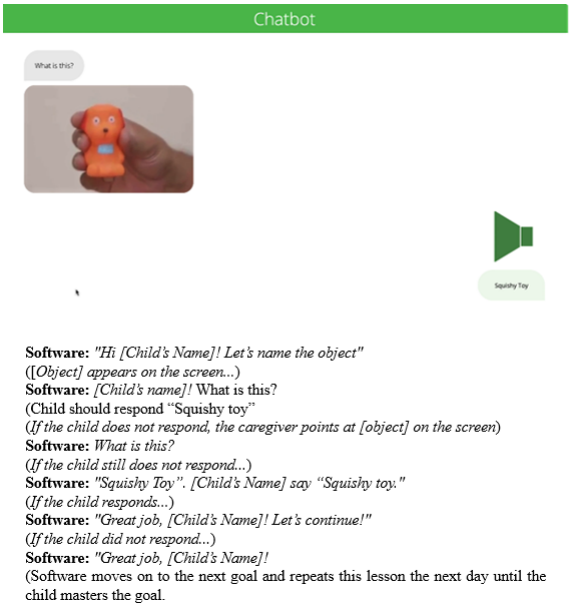
A screenshot of a lesson and an example transcript of a child-software interaction.

Once the child achieved 3 goals, a new ILP with a new set of 3 goals was created. To achieve each goal, the child is taught through 4 modalities:

- Audiovisual stimulation: Concepts are introduced through video modeling with interactive questions embedded within the content, increasing with complexity across four levels (level 0, 1, 2, and 3). Prompts are provided to guide the child’s learning and are gradually reduced as the child becomes more proficient.
- Chatbot: This feature uses interactive questions to reinforce learning and promote generalization. The feature is particularly effective in fostering verbal engagement and enhancing the child’s communication skills. An example of a chatbot goal is given in Figure 3.
- AI-based interactive games: Learning is facilitated through AI-driven interactive games that are tailored to each child’s learning style, making the learning engaging and adaptive to individual needs.
- Home-based parent training videos: To support home-based activities, parents are provided with instructional videos that demonstrate how to apply the skills learned by their child in various settings, thus reinforcing learning outside the therapy center. The child’s performance is assessed using 3 metrics captured by the software: first-time rights (accuracy of initial responses), correct questions (total number of correctly answered questions), and number of questions attempted (total engagement with the learning material). Once the lesson is mastered, the software automatically assigns the next set of goals.

If a child is not progressing toward their goals, the system proactively alerts the parents and therapists. Separately, parents are instructed to record a video of the lesson and submit it to the study coordinator or therapist team for review. In response, therapists will simplify the web-based goals to better suit the child’s needs. Should the child continue to struggle, parents will receive a notification prompting them to resubmit the ILP checklist. Following this, the system will reassign 3 new goals, which will be carefully verified by therapists to ensure they align with the child’s learning trajectory.

### Other Core Features of the Platform

Other core features of the CognitiveBotics platform include the following:

- ILP progression: The software adjusts the level of difficulty of the ILP based on the child’s progress, providing necessary assistance and notifications to parents and therapists.
- Personalization: Personalization is a unique feature, where all learning goals are delivered in a personalized and customized manner, tailored to the specific needs of each child. During interactive sessions, the system personalizes by using the child’s name while asking the interactive questions, drawing the child’s attention.
- Dashboard: A daily progress graph is displayed on the child’s dashboard, which is accessible to both parents and therapists, offering real-time insights into the child’s development.
- Two-way communication: The software includes a fun activity that detects and encourages body part interactions, in addition to occupational therapy tasks, promoting overall development from a young age.
- Objective videos: Parents are empowered through videos that outline the objectives of each task, enabling them to actively participate in and support their child’s learning.
- Data capture and progress tracking features: Aim to automate monitoring and capture the child’s progress based on key learning principles—attention, retention, and generalization, such as “eye gaze detection.” These data are presented in a user-friendly format on a dashboard, facilitating easy comprehension for both parents and therapists.

### Fidelity of Implementation Data

The fidelity of implementation was assessed via a multitiered approach to ensure attendance to the session lessons. The software has an automated session notification and progress tracker to prompt parents to complete assigned goals within the learning plan. To progress to the next learning level, mandatory successive mastering of goals is required. This ensures that all lesson components were completed as intended. Additionally, therapist-led monitoring and follow-up calls were conducted to monitor progress, reinforce engagement with the intervention, and address any caregiver-reported concerns to ensure fidelity.

Caregivers underwent a structured training program on reinforcement strategies aimed at ensuring consistency in their interactions with the child beyond software-guided sessions. This training equipped caregivers with evidence-based behavioral techniques that align with the principles of ABA and developmental learning models, such as immediate reinforcement or reward systems. Furthermore, to encourage parental involvement, caregivers were provided zero-fee in-person therapy sessions at the center, on the condition their child is actively engaged with the platform.

Lastly, software usage was collected at the back end, tracking metrics such as log-in frequency, time spent on lessons, and completion rates. This allowed the software programmer to evaluate the platform utilization and adherence. Any deviations from the lesson plans were brought to the attention of the therapist. Together, these mechanisms ensured consistent implementation and provided opportunities for timely intervention when necessary.

### Statistical Analysis

After completion of the study, the data were analyzed to compare the effectiveness of the CognitiveBotics platform between the intervention and control groups. For each group and clinical assessment parameter, the mean scores and standard deviations were calculated at 2 stages: the start of the study (T1) and the end of the study (T2). The mean change and percentage mean change from T1 to T2 were also computed. To determine the statistical differences, the *P* values were calculated using the Mann-Whitney *U* test, with a *P* value of <.05 being considered as statistically significant.

### Ethical Considerations

This study was conducted in accordance with the study protocol, the New Drugs and Clinical Trials Rules 2019 issued by the Government of India, the ethical principles that have their origin in the Declaration of Helsinki (64th World Medical Association General Assembly, Fortaleza, Brazil, October 2013), the International Council for Harmonisation Good Clinical Practice, and all applicable local regulatory requirements. The investigators agreed to conduct the study according to the principles of the International Council for Harmonisation Good Clinical Practice, as well as in accordance with the ethical principles that have their origin in the Declaration of Helsinki, the protocol, and all national, state, and local laws or regulations. The medical care given to and medical decisions made on behalf of study participants were always the responsibility of a principal (site) investigator. Each individual involved in conducting the study was qualified by education, training, and experience to perform his or her respective task(s).

Informed consent was obtained from the parents or legal guardians of all participants. The study details were thoroughly explained, including the study’s purpose and procedures and the voluntary nature of participation. Parents were informed that they and their children were free to withdraw from the study at any time, with no impact on their routine activities or any other services received. As this study included human participants, the collection of data from medical records, as well as software usage, it adheres to all institutional ethical guidelines. Ethical approval for this observational study was obtained from the Institutional Ethics Committee of the Rainbow Children’s Medicare (registration number EC/RENEW/INST/2021/10510).

Before any collection of data, the study protocol, participant information sheets, and informed consent forms were reviewed and approved. The data were maintained throughout the study, with all reports and communications relating to participants being kept confidential. Names and other identifiable details were removed, and all records were coded using unique identification acronyms. No images or video recordings of participants are included in the manuscript. No monetary compensation was provided to the participants or their families. However, participants in both the intervention and control groups received free access to the software platform, as well compensation for travel expenses when coming to the center for assessments.

## Results

### Participant Selection and Characteristics

The results of this study examine the impact and utility of the CognitiveBotics platform for children with ASD over a 12-month observational period. Key outcomes focus on quantitative measures of behavioral, developmental, and language-based parameters. An intervention versus control analysis was performed, organized by baseline (T1) and end-of-study (T2), to ascertain the software’s impact across multiple functional and developmental domains, namely CARS, VSMS, DST, and REEL scores. This approach provided structured insights into the software’s influence on each parameter and allowed for comparative analysis of outcomes over time.

**Figure 4.**
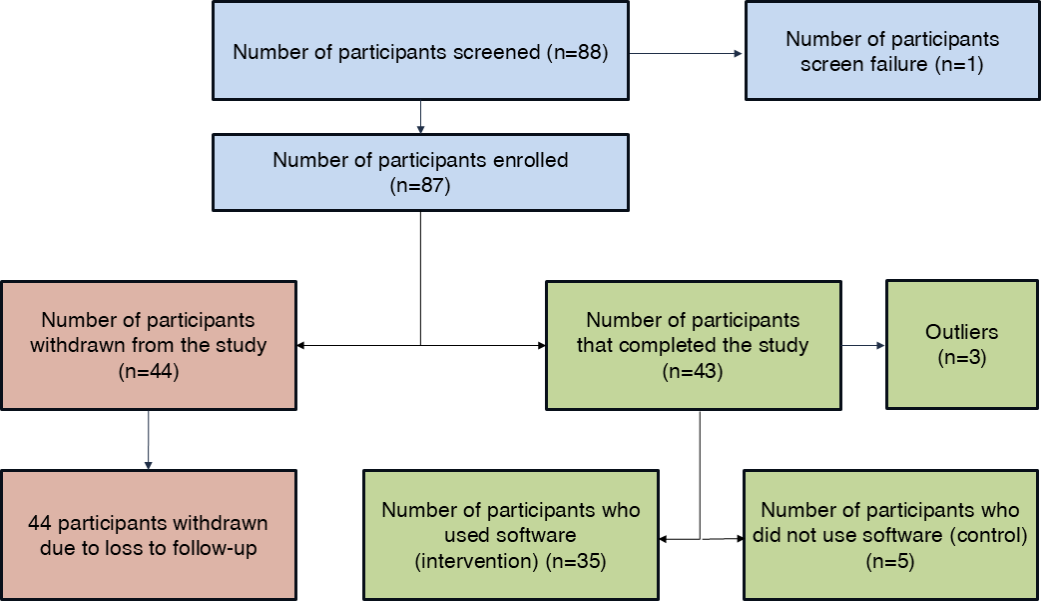
Flowchart of participants in the study.

Figure 4 illustrates the study’s recruitment and retention flow. Of an initial total of 88 enrolled participants, 43 completed the study, while 35 continued to use the software for the entire 1-year duration, and 5 did not use the software but participated in the 1-year follow-up assessments, and were categorized as the control group. A further 3 participants were labeled as outliers and were excluded from further analysis. Table 2 shows the key baseline demographic characteristics of the 40 participants who completed the study.

**Table 2.** Comparison of baseline demographics of participants in the intervention and control groups.

Parameter and statistics		Intervention (n=35)	Control (n=5)	Overall (n=40)
**Age (years)**				
	Mean (SD)	43.71 (SD 15.48)	44.60 (SD 14.98)	43.83 (SD 15.23)
	Median	39.00	39.00	39.00
	Quantile	31.50; 52.00	33.00; 54.00	31.75; 54.50
	Range	25.00‐87.00	31.00‐66.00	25.00‐87.00
**Gender, n (%)**				
	Male	33 (94)	3 (60)	36 (90)
	Female	2 (6)	2 (40)	4 (10)

The participants in the intervention group were stratified into 3 developmental groups based on chronological age:

- Toddler group (n=12): children aged 2‐3 years
- Preschool group (n=15): children aged 4‐6 years
- School-aged group (n=8): children aged 7‐8 years

The purpose was to assess the impact of the intervention across different developmental ages, considering variations in cognitive, language, and social skills.

Based on the study location, the majority of participants were of South Indian descent and from families with a higher educational background. All participants showed delays across multiple developmental domains, necessitating structured therapeutic intervention. Their academic skill levels in reading, writing, and mathematics were rudimentary, with significant challenges observed in social/emotional, language/communication, cognitive, and movement/physical development.

### Intervention and Control Group–Based Analysis Using Different Parameters

The study evaluated outcome measures in the intervention and control groups across T1 (baseline) and T2 (12 months), assessing CARS, SA, SQ, DA, DQ, and REEL scores.

Table 3 shows the outcome measures of 35 participants in the intervention group, which were compared across T1 and T2. For the CARS score, there was a significant decrease from 33.41 (SD 1.89) at T1 to 28.34 (SD 3.80) at T2, showing a mean change of 5.07 and a percentage change of 15.18% (*P*<.001).

**Table 3.** Comparison of outcome measures in the intervention group only at baseline (T1) and end of study (T2).

Parameters	Intervention group (n=35**)**				
	T1^a^, mean (SD)	T2^b^, mean (SD)	Mean change	Mean change, %	*P* value^c^
CARS^d^	33.41 (1.89)	28.34 (3.80)	5.07	15.18	<.001
SA^e^	22.80 (7.33)	35.76 (9.09)	12.96	56.84	<.001
SQ^f^	53.26 (11.84)	64.75 (16.12)	11.49	21.57	<.001
DA^g^	30.93 (9.91)	45.31 (11.20)	14.38	46.49	<.001
DQ^h^	70.94 (10.95)	81.33 (16.85)	10.39	14.65	<.001
RLA^i^	22.09 (8.94)	34.51 (14.93)	12.42	56.22	<.001
ELA^j^	18.69 (8.52)	29.89 (15.60)	11.20	59.93	<.001

a T1: start of the study.

b T2: end of the study.

c *P* value was calculated using the Mann-Whitney *U* test.

d CARS: Childhood Autism Rating Scale.

e SA: social age.

f SQ: social quotient.

g DA: developmental age.

h DQ: developmental quotient.

i RLA: receptive language age.

j ELA: expressive language age.

In the SA score, there was a significant improvement from 22.80 (SD 7.33) at T1 to 35.76 (SD 9.09) at T2, with a mean change of 12.96 and a percentage change of 56.84% (*P*<.001).

In the SQ score, there was an improvement from 53.26 (SD 11.84) at T1 to 64.75 (SD 16.12) at T2, with a mean change of 11.49 and a percentage change of 21.57% (*P*<.001).

In the DA score, there was an improvement from 30.93 (SD 9.91) at T1 to 45.31 (SD 11.20) at T2, showing a mean change of 14.38 and a percentage change of 46.49% (*P*<.001).

In the DQ score, there was an improvement from 70.94 (SD 10.95) at T1 to 81.33 (SD 16.85) at T2, showing a mean change of 10.39 and a percentage change of 14.65% (*P*<.001).

In the REEL score, the RLA showed a substantial increase from 22.09 (SD 8.94) at T1 to 34.51 (SD 14.93) at T2, with a mean change of 12.42 and a percentage change of 56.22% (*P*<.001). Similarly, the ELA exhibited a significant increase from 18.69 (SD 8.52) to 29.89 (SD 15.60), showing a mean change of 11.20 and a percentage change of 59.93% (*P*<.001).

Table 4 shows the outcome measures of 5 participants in the control group, which were compared across T1 and T2. For the CARS score, there was a significant decrease from 33.90 (SD 1.24) at T1 to 30.30 (SD 3.68) at T2, showing a mean change of 3.6 and a percentage change of 10.62% (*P*=.06).

**Table 4.** Comparison of outcome measures in the control group only at baseline (T1) and end of study (T2).

Parameters	Control group (n=5)				
	T1^a^, mean (SD)	T2^b^, mean (SD)	Mean change	Mean change, %	*P* value^c^
CARS^d^	33.90 (1.24)	30.30 (3.68)	3.6	10.62	.06
SA^e^	21.41 (5.44)	32.60 (8.24)	11.19	52.27	.06
SQ^f^	49.13 (5.45)	58.77 (14.73)	9.64	19.62	.12
DA^g^	28.30 (6.69)	41.00 (7.04)	12.7	44.88	.06
DQ^h^	65.60 (11.68)	72.97 (7.22)	7.37	11.23	.19
RLA^i^	19.60 (7.13)	26.40 (9.53)	6.80	34.69	.10
ELA^j^	16.80 (4.60)	23.60 (6.23)	6.80	40.48	.054

a T1: start of the study.

b T2: end of the study.

c *P* value is calculated using Mann-Whitney *U* test.

d CARS: Childhood Autism Rating Scale.

e SA: social age.

f SQ: social quotient.

g DA: developmental age.

h DQ: developmental quotient.

i RLA: receptive language age.

j ELA: expressive language age.

In the SA score, there was a significant improvement from 21.41 (SD 5.44) at T1 to 32.60 (SD 8.24) at T2, with a mean change of 11.19 and a percentage change of 52.27% (*P*=.06).

In the SQ score, there was an improvement from 49.13 (SD 5.45) at T1 to 58.77 (SD 14.73) at T2, with a mean change of 9.64 and a percentage change of 19.62% (*P*=.12).

Similarly, in the DA score, there was an improvement from 28.30 (SD 6.69) at T1 to 41.00 (SD 7.04) at T2, showing a mean change of 12.7 and a percentage change of 44.88% (*P*=.06).

In the DQ score, there was an improvement from 65.60 (SD 11.68) at T1 to 72.97 (SD 7.22) at T2, showing a mean change of 7.37 and a percentage change of 11.23% (*P*=.19).

In the REEL score, the RLA showed a substantial increase from 19.60 (SD 7.13) at T1 to 26.40 (SD 9.53) at T2, with a mean change of 6.80 and a percentage change of 34.69% (*P*=.10). The ELA exhibited an increase from 16.80 (SD 4.60) to 23.60 (SD 6.23), showing a mean change of 6.80 and a percentage change of 40.48% (*P*=.054).

Overall, the intervention group presented substantial improvements across all outcome measures, particularly in CARS, SA, and language scores (RLA and ELA), with the majority of these changes reaching statistical significance. This indicates that the platform may enhance social, cognitive, and language outcomes in the intervention group. In contrast, the control group of 5 participants showed positive changes but with less significance and the changes were statistically weaker across measures.

## Discussion

### Principal Findings

This study demonstrated that CognitiveBotics, an AI-powered assistive technology, has made significant gains in developmental and social parameters over the course of 12 months in children diagnosed with autism. Both parents and therapists have reported minimal negative behavioral changes while using the platform, including screen addiction and sleep disturbances. In intervention versus control analysis, there were significant improvements in the intervention group, particularly in those with higher baseline levels of functioning, underlining the efficacy of the software in reducing autism severity and enhancing developmental skills in children with ASD. Accompanied by highly significant *P* values, the intervention group showed an improvement in symptoms, as well as marked enhancements in social skills, developmental age, and language abilities.

The CognitiveBotics software, like many other available ABA-assistive technologies, was observed to have various benefits and advantages specifically for individuals with ASD [50]. Supported in laptops and tablets, the platform is commonly available, affordable, and socially acceptable, making it an ideal tool for parent-mediated interventions [51,52]. Using the platform, parents played a crucial role in supporting their children’s learning, observing better improvements compared to the control group using only traditional therapy. The software helps enhance attention span and motivation during learning activities, offering engaging, interactive experiences that increase children’s participation [53,54].

Within a learning environment, the software increases interaction and participation and improves the learning process [55]. Additionally, the software provides real-time feedback on key skills and is customizable to focus on individual needs, similar to the benefits seen in the Picture Exchange Communication System and other visual aids, texts, and sounds [56,57]. The portability of the devices can allow parents to provide learning at times when the child is most receptive, despite the unavailability of therapists. Furthermore, parent-implemented technologies can be the most readily and affordably deployed, and such assistive technology enables parents to offer the most opportunities for social contact [58]. The software incorporates interactive games that improve social-emotional functioning and behavior. The interactive feature allowed the participants to recognize emotions, use deconfliction strategies, collaborate with others, and address issues like greeting known people like teachers or neighbors. In a recent study, parents who used social skills programs incorporating features similar to those in the CognitiveBotics platform found significant improvements in social skills and reductions in problematic behaviors, in contrast to those in the control group [59].

There may be certain shortfalls with the use of ABA assistive technologies, but as with any problem, there are solutions that can overcome such shortfalls. The first area of concern is increased screen time, possibly leading to restricted or repetitive behaviors, lack of socializing, and concerns over metabolic and sleep disturbances [60,61]. In such circumstances, CognitiveBotics has incorporated a preset screen time feature of 20 minutes, after which the session concludes and takes the user to the dashboard. It is also advisable to provide minimal access in a group setting to reduce potential isolation [62]. Devices may also be misused to view passive content, in which case supervised coviewing with parents is advised [63]. Furthermore, the choice of content has to be predetermined, whereby highly interactive and engaging media is most beneficial to the child as it promotes engagement, motivation, and learning outcomes [64]. Another issue is the potential for tantrums if the device is removed. As is the case in other situations, when access to preferred items is interrupted, parents and therapists should be trained to control such behaviors.

In recent years, there have been numerous studies on the proposed use of tablets or computers in autism interventions. A meta-analysis conducted by Sandbank et al [65], reviewed 252 separate trials examining the efficacy of technology in autism interventions. The findings suggest an overall improvement in social communication skills and reductions in difficult behaviors, particularly when used by parents. This aligns with the intentions behind the CognitiveBotics platform, which aims to support individuals with autism and their families. Furthermore, a low incidence of adverse events reported when using such interventions supports adoption of the software in both home and clinical settings.

Novack et al [66] conducted a study to assess the effectiveness of mobile apps on the principles of ABA, particularly in assessing the impact on the receptive language skills of individuals. Randomized into an immediate-treatment or a delayed-treatment control group, the results indicated significant improvements in receptive language skills in the former group. However, the study had limitations, particularly with the absence of psychometric parameters to assess outcomes. Although improvements in receptive language skills were observed, the study is incomplete. Our 12-month study demonstrated how CognitiveBotics leverages AI to improve receptive language skills, offering prolonged benefits using personalized ABA-based interventions and addressing limitations in traditional psychometric assessments. Another study aimed at addressing social engagement by using a proposed 3D complex facial expression recognition system to recognize facial emotions; it found that, in 3 weeks, users had a marked improvement in identifying facial cues compared with the control group, with surprise and shy expressions being the easiest to identify [67]. Similarly, CognitiveBotics contains activities that enable children to better recognize and respond to social and emotional cues, significantly boosting their social communication skills within a short intervention period.

A study conducted in Saudi Arabia assessed the effectiveness of AI-driven apps in a traditional education setup. Apps such as “My School” and “Alfaz” were chosen for their adaptive and interactive content that aligned with the academic curriculum. Participants who received 60-minute sessions twice weekly for 5 weeks showed significant improvements in reading and math skills compared to those in the control group [68]. Similarly, our software incorporates real-time feedback, task adaptation, and data-driven insights to ensure that children receive targeted, engaging, and effective support, ultimately enhancing their cognitive and functional independence.

Lastly, a meta-analysis conducted by Moon et al [23] aimed to review the effectiveness of mobile apps in the treatment of individuals with ASD. After a review of 1100 randomized controlled trials, only 7 studies were deemed suitable for further analysis, suggesting a very methodological approach. Using the Mullen Scales of Early Learning, the results favored the intervention group, indicating a significant improvement in the participants’ early learning and developmental outcomes compared to control groups. Moreover, the analysis found minimal heterogeneity (*P*>.10) across different studies or no significant evidence of publication bias. Correspondingly, our platform aligns with these findings by offering a technology-based, interactive tool specifically designed to enhance learning and developmental progress in individuals with autism. With an emphasis on providing individualized interventions that target key skills, CognitiveBotics uses validated clinical parameters to monitor improvements, reducing inaccuracies, similar to the studies highlighted in Moon’s analysis [23].

### Limitations of the Study

Although evidence from our longitudinal study shows significant improvement in outcome measures for individuals with ASD using the software, a few limitations have to be discussed. First, the small sample size of 40 participants is a critical limitation, suggesting inadequate generalization of the findings. However, most studies regarding children with autism often face challenges in recruiting adequate numbers of participants. Limited research has explored effective strategies for efficiently recruiting participants with ASD, a challenge that poses a barrier to larger and more comprehensive studies in this field [69].

Second, the participants were recruited from a single center and predominantly came from literate and urban families. Such a demographic is not representative of the entire population of individuals with ASD, particularly in India. The benefits observed in using the software may not translate to individuals with a lower socioeconomic status or those located in rural areas, who may face different challenges and have different needs. Further studies should be conducted to include participants from rural areas and various socioeconomic backgrounds. This includes incorporating features that reflect local languages and cultural sensitivities to ensure the software is relevant and effective for a wider range of users.

Third, the study experienced a 59% attrition rate, which could be attributed to several factors, including language barriers or the demanding schedules of caregivers, which may have limited their ability to fully engage with the platform. Such high levels of attrition are commonly observed in digital therapeutics for mental health. Similarly, a recent meta-analysis found more than half of the users discontinued using smartphone apps aimed at treating depressive symptoms [70].

Finally, while randomized controlled trials are considered the gold standard for assessing the effectiveness of interventions, their feasibility in such a population remains challenging. To address this, future research should explore methodologies that balance scientific rigor with practical implementation to further validate the software’s effectiveness among different subgroups.

### Conclusions

This 12-month study demonstrated that the CognitiveBotics platform delivering parent-mediated interventions significantly improved multiple developmental and social parameters in participants. Furthermore, it highlights that these digital technologies using audiovisuals, AI-based interactive games, animation games, and chatbots have an attraction factor that keeps the interest of children with ASD. Particularly, the incorporation of AI into digital technology has been shown to enhance social communication skills, especially in younger participants with learning difficulties, helping them reach their specific learning objectives.

Most assistive technologies are not intended to satisfy the needs of individuals with ASD as a whole, as they have variable needs. Despite being in its infancy, such digital technologies have been proposed to address the wide array of learning needs and work on the core symptoms of ASD. Further research must be conducted to include a larger number of children with different levels of social and developmental delays and ASD severity along with regional, linguistic, and sociocultural variations.

In conclusion, the promising results of this study underscore the potential of AI software interventions in revolutionizing holistic support for children with ASD. As these technologies continue to evolve, aligning the software not just to the needs of the child but also to those of parents and therapists offers hope for more personalized and effective strategies for not just children on the autism spectrum but also all neurodiverse children.

## Abbreviations

ABA: applied behavioral analysis
AI: artificial intelligence
ASD: autism spectrum disorder
CAI: computer-assisted interventions
CARS: Childhood Autism Rating Scale
DA: developmental age
DQ: developmental quotient
DST: Developmental Screening Test
ELA: expressive language age
ILP: individualized learning plan
REEL: Receptive and Expressive Emergent Language Test
RLA: receptive language age
SA: social age
SQ: social quotient
VSMS: Vineland Social Maturity Scale
